# Exaggerated Pressor Response in Relation to Attenuated Muscle Temperature Response during Contraction in Ischemic Heart Failure

**DOI:** 10.3389/fphys.2012.00443

**Published:** 2012-11-26

**Authors:** Jianhua Li, Zhaohui Gao, Jian Lu, Jihong Xing

**Affiliations:** ^1^Heart and Vascular Institute, Milton S. Hershey Medical Center, Pennsylvania State University College of MedicineHershey, PA, USA; ^2^Department of Medicine, Milton S. Hershey Medical Center, Pennsylvania State University College of MedicineHershey, PA, USA

**Keywords:** heart failure, exercise, muscle temperature, blood pressure, sympathetic nervous system

## Abstract

It is known that muscle temperature (*T*_m_) increases with exercise. The purpose of this study was to examine if contraction-induced increase in *T*_m_ was altered in rats with heart failure (HF) induced by chronic myocardial infraction (MI) as compared with healthy control animals. A temperature probe was inserted in the triceps surae muscle to continuously measure *T*_m_ throughout experiments. Static muscle contraction was induced by electrical stimulation of the sciatic nerve for 1 min. As baseline *T*_m_ was 34°C, contraction increased temperature by 1.6 ± 0.18°C in nine health control rats and by 1.0 ± 0.15°C in 10 MI rats (*P* < 0.05 vs. control). Note that there were no differences in developed muscle tension and muscle weight between the two groups. In addition, muscle contraction increased mean arterial pressure by 23 ± 3 mmHg in control rats and by 31 ± 3 mmHg in MI rats (*P* < 0.05 vs. control). A regression analysis further shows that there is an inverse liner relationship between the pressor response and static contraction-induced increase in *T*_m_. Our data suggest that *T*_m_ increase evoked by contraction is impaired in MI rats. The abnormal alteration in *T*_m_ likely modifies the reflex cardiovascular responses in MI via mechanisms of temperature-sensitive receptors on muscle afferent nerves.

## Introduction

The sympathetic nervous system is activated during exercise (Mark et al., 1985; Victor et al., 1988; Sinoway et al., 1989; Matsukawa et al., 1990). This contributes to increases in blood pressure, heart rate (HR), myocardial contractility, and peripheral vasoconstriction (Coote et al., 1971; McCloskey and Mitchell, 1972; Mitchell et al., 1977; Lind, 1983). Two mechanisms of neural control contribute to these exercise responses: “central command” and “the exercise pressor reflex” (Goodwin et al., 1972; Mitchell et al., 1983; Waldrop et al., 1996). Central command is a mechanism whereby signals from a central site responsible for recruiting motor units activate cardiovascular control areas in the brain stem (Goodwin et al., 1972; Waldrop et al., 1996). The exercise pressor reflex is a mechanism whereby signals from thin-fiber skeletal muscle group III (predominately mechanically sensitive) and group IV (predominately metabolically sensitive) afferents likewise evoke increases in blood pressure and HR via coordinated changes in autonomic outflow (Kaufman et al., 1983; Mitchell et al., 1983; Kaufman and Forster, 1996). This system responds to mechanical deformation of the muscle afferent receptive field (i.e., “mechanoreceptor” stimulation) as well as to metabolic stimulation (i.e., “metaboreceptor” stimulation). When these receptors are stimulated, thin-fiber muscle afferent nerves are engaged, cardiovascular circuits in the brain stem are activated, sympathetic activity increases, and blood pressure rises (Mitchell et al., 1983).

The sympathetic nerve and blood pressure responses to exercise are exaggerated in heart failure (HF; Middlekauff et al., 2000, 2001; Li et al., 2004b; Momen et al., 2004; Gao et al., 2007). However, the underlying mechanisms to cause the abnormal autonomic responses are poorly understood.

In general, purinergic P2X receptors and transient receptor potential vanilloid type 1 (TRPV1 or VR1) are widely found on thin-fiber afferent nerves (Guo et al., 1999; Ma, 2002) and mediates numerous sensory afferent activations (Caterina et al., 1997; Nault et al., 1999; Smith and McQueen, 2001; Zahner et al., 2003). Specifically, it has been reported that activation of P2X receptors on the nerve endings of muscle afferents plays a role in mediating the autonomic adjustments to active muscle (Hanna et al., 2002; Li and Sinoway, 2002; Hanna and Kaufman, 2003, 2004). Although TRPV1 has been reported to play little role in mediating the cardiovascular responses to activation of muscle afferent (Kindig et al., 2005), capsaicin, a TRPV1 agonist injected into the arterial supply of the hindlimb muscles evokes increases in blood pressure and HR (Li et al., 2004a,b; Smith et al., 2005).

Furthermore, abnormal responses of P2X and TRPV1 have been observed in rats with HF. For instance, previous studies have demonstrated that muscle afferent-mediated pressor response of P2X activation is exaggerated in HF animals and the responsiveness is related to the degree of left ventricular dysfunction (Li et al., 2004b; Gao et al., 2007). The augmented reflex response is likely linked to upregulated P2X receptors in the sensory neurons of thin-fiber afferent nerves in HF (Gao et al., 2007; Wang et al., 2010). In addition, less TRPV1 expression is found in the sensory neurons of HF rats compared with control animals, and the pressor response of TRPV1 activation is attenuated in HF (Li et al., 2004b; Smith et al., 2005; Wang et al., 2010).

Muscle temperature (*T*_m_) rises in exercising muscles (Shellock et al., 1985; Kenny et al., 2003), and P2X and TRPV1 are sensitive to change of temperature (Garcia-Villalon et al., 1997; Wang et al., 2003; Kluess et al., 2005). Thus, it was necessary to study contraction-induced increase in *T*_m_ in order to better understand heightened sympathetic activity in HF, likely due to P2X and TRPV1. Moreover, the published data have demonstrated that blood pressure response to stimulation of P2X receptors on muscle afferents is attenuated with increasing *T*_m_ (Gao et al., 2006). This result suggests that higher *T*_m_ blunts effects of P2X on the reflex pressor response. Likewise, lower *T*_m_ augments effects of P2X. In contrast, higher temperature increases its response as TRPV1 is activated (Caterina et al., 1997). Given that P2X is increased and TRPV1 is decreased after induction of myocardial infraction (MI; Smith et al., 2005; Gao et al., 2007), we hypothesized that contraction-induced increase in *T*_m_ is attenuated in HF rats and then abnormal *T*_m_ response in HF may affect the muscle pressor reflex via temperature-sensitive P2X and TRPV1 (Garcia-Villalon et al., 1997; Wang et al., 2003; Kluess et al., 2005). Moreover, a relationship between the reflex pressor response and *T*_m_ response during contraction was further determined in this report.

## Materials and Methods

### Coronary artery ligation

All procedures outlined in this study were approved by the Animal Care Committee of this institution. Sprague Dawley male rats (150–180 g) were anesthetized by inhalation of isoflurane oxygen mixture (2–5% isoflurane in 100% oxygen), intubated, and artificially ventilated. A left thoracotomy between the fourth and fifth ribs was performed, exposing the left ventricular wall. The left coronary artery was ligated. Experiments were performed 6–10 weeks after coronary ligation. Age- and body weight-matched rats served as controls.

Transthoracic echocardiography was performed 1–2 weeks before the experiments. The rats were anesthetized by inhalation of isoflurane oxygen mixture (2–5% isoflurane in 100% oxygen). The transducer was positioned on the left anterior chest, and left ventricular dimensions were measured. The fractional shortening (FS) was determined by echocardiographic measurements. FS is >40% in controls (*n* = 9) and <30% in HF (*n* = 10), respectively.

### Experimental preparation

The rats were anesthetized by inhalation of isoflurane oxygen mixture (2–5% isoflurane in 100% oxygen). An endotracheal tube was inserted into the trachea and attached to a ventilator. Polyethylene catheters (PE-50) were inserted into the common carotid artery and external jugular vein for measurement of arterial blood pressure and for fluid administration, respectively. The skin covering the triceps surae muscle and femoral region was surgically separated from the muscle below in order to eliminate inputs from cutaneous afferents in the hindlimb. The sciatic nerve of each leg was isolated and then placed on a stimulating electrode. A needle microprobe connected to a thermometer (Model BAT-12, World Precision Instrument, Sarasota, FL, USA) was directly inserted into the gastrocnemius muscle of the hindlimb to continuously monitor baseline *T*_m_ throughout experiments and measure *T*_m_ responses to muscle contraction. The animals were ventilated, and tidal CO_2_ was monitored by a respiratory gas monitor (Datex-Ohmeda, Madison, WI, USA) and maintained within normal ranges, as previously described (Li et al., 2004a,b; Gao et al., 2007). Body temperature was maintained between 36.5 and 38.5°C by a heating pad and external heat lamps, and fluid balance was stabilized by a continuous infusion of saline.

Decerebration was performed as previously described (Li et al., 2004a,b; Smith et al., 2005; Gao et al., 2007). A transverse section was made anterior to the superior colliculus. Once this procedure was completed, anesthesia was removed from the inhaled mixture. A recovery period of 60 min after decerebration was employed to allow sufficient time for elimination of the effects of anesthesia gas from the preparation.

Arterial blood pressure was measured by connecting the carotid arterial catheter to a pressure transducer (model P23ID, Statham). Mean arterial pressure (MAP) was obtained by integrating the arterial signal with a time constant of 4 s. HR was determined from the arterial pressure pulse. All measured variables were continuously recorded on an eight-channel chart recorder (model TA 4000, Gould, Valley View, OH, USA) and stored on a PC computer that used the PowerLab system (ADInstruments, Castle Hill, Australia). The triceps surae muscle was isolated and the calcaneal bone of the hindlimb was cut. The Achilles tendon was connected to a force transducer for the measurement of muscle tension during electrically induced muscle contraction. The pelvis was stabilized in a spinal unit and the knee joints were secured by clamping the patellar tendon to a spinal unit.

On completion of each experiment, a 2-Fr microMillar pressure transducer catheter (Millar Instruments) was inserted into the right carotid artery and threaded into the left ventricle for measurement of left ventricular end-diastolic pressure (LVEDP). The heart was exercised after intravenous injection of an overdose of sodium pentobarbital (120 mg/kg body weight) followed by 2 ml of a saturated solution of potassium chloride. Wet heart and triceps surae muscle weight were measured. The data collected from rats whose left ventricle FS was <30% were included in MI group of this report.

### Experimental protocols

A 60 min equilibration period was allowed after completion of experimental procedure. Contractions induced by electrical stimulation of the sciatic nerve were then performed in nine healthy control rats and 10 MI rats. Static contraction of the triceps surae muscle was conducted at frequencies of 30 Hz (2.5 times motor threshold and 0.1 ms duration; Gao et al., 2006). Stimulation was sustained for 1 min. Baseline *T*_m_ was controlled at 30, 34, and 38°C at a random way, respectively, by using a water-perfused heating pad and an ice bag around the hindlimb muscle. Contraction was performed at different baselines of *T*_m_. There was a 60 min rest period between each bout of contraction. The *T*_m_ was measured before, during, and after each of stimulations. Body core temperature and *T*_m_ on the contralateral leg were also measured during stimulation.

### Data acquisition and analyses

Arterial blood pressure and developed muscle tension during muscle contraction were recorded on a PC computer that used Power Lab software. *T*_m_ was recorded on a temperature monitor. Control values were determined by averaging at least 1 min of the data immediately before the interventions. The peak change of each variable was determined by the peak response from control.

Peak change data for each variable were analyzed with a two-way ANOVA. Changes in temperature during 1 min-stimulation in control rats and MI rats were also analyzed with a two-way ANOVA. Tukey *post hoc* analyses were utilized to determine differences between groups, as appropriate. All values were expressed mean ± SE. For all analyses, differences were considered significant at *P* < 0.05. All statistical analyses were performed using SPSS for windows version 13.0.

## Results

Rats with the left ventricular FS < 30% showed increases in heart weight, LVEDP, and left ventricular diastolic dimension (Table 1). These rats were used as a MI group. In addition, there were no significant differences in resting *T*_m_ and core body temperature as well as body and muscle weight between control and MI groups (Table 2).

**Table 1:** **Echocardiographic and cardiac characteristics**.

	LVAW (cm)	LVDD (cm)	LVPW (cm)	LVSD (cm)	FS (%)	Heart weight (g)	LVEDP (mmHg)
Control (*n* = 9)	0.14 ± 0.01	0.89 ± 0.01	0.15 ± 0.01	0.40 ± 0.02	55.32 ± 2.14	1.42 ± 0.02	0.4 ± 0.18
HF (*n* = 10)	0.08 ± 0.00*	1.09 ± 0.02*	0.16 ± 0.01	0.87 ± 0.02*	19.74 ± 1.33*	1.79 ± 0.04*	15 ± 3.00*

LVAW, the thickness of left ventricular anterior wall; LVDD, left ventricular end-diastolic dimension; LVPW, left ventricular posterior wall; LVSD, left ventricular end-systolic dimension; FS, shortening fraction of LV; LVEDP, LV end-diastolic pressure. Values are mean ± SE. *indicates, *P* <  0.05, vs. control.

**Table 2:** **Muscle characteristics**.

	Number of rats	Body weight(BW, g)	Muscle weight (MW, g)	MW/BW (mg/g)	Basal body temperature (°C)	Basal muscle temperature (°C)
Control	9	535 ± 19.4	3.69 ± 0.081	6.9 ± 0.1	36.83 ± 0.20	34.50 ± 0.16
HF	10	515 ± 9.28	3.67 ± 0.078	7.1 ± 0.1	36.99 ± 0.14	34.43 ± 0.11

*Muscle indicates the triceps surae muscle. Values are mean ± SE. There are no differences in all parameters between the two groups*.

### *T*_m_ response in control and MI rats

**Figure 1:**
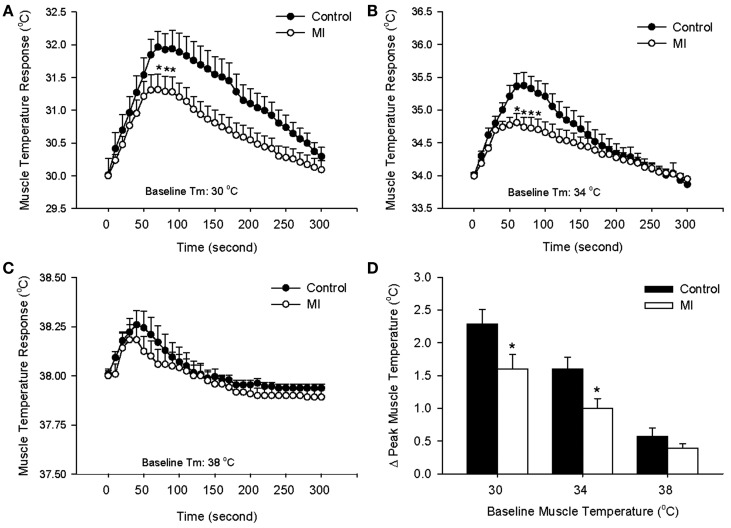
**Contraction-induced increase in muscle temperature (*T*_m_) in control rats and MI rats during 1 min electrical stimulation of sciatic nerve and 4 min follow-up at different basal *T*_m_: 30°C (A), 34°C (B), and 38°C (C)**. The peak temperature changes in the muscle during static contraction at three levels of basal *T*_m_ are shown in **(D)**. Values are means ± SE (Control: 9 rats; MI: 10 rats). The increases in *T*_m_ during contraction were significantly lower in MI at baseline *T*_m_ of 30, 34°C. *P < 0.05, compared with healthy control group.

Following a start of electrical stimulation of the sciatic nerve, *T*_m_ increased. The response gradually returned to a steady level after the end of stimulation. At baseline *T*_m_ of 30, 34, and 38°C, time courses of contraction-induced increase in *T*_m_ during 1 min of contraction are shown in Figures 1A–C. Peak temperature responses to contraction in control rats and MI rats are shown in Figure 1D. It is noted that an increase in *T*_m_ was significantly attenuated in MI rats compared with control rats, as resting *T*_m_ was 30 and 34°C. However, this effect wasn’t observed, as resting *T*_m_ was 38°C. Body core temperature and *T*_m_ on the contralateral leg were not significantly changed during stimulation.

### Cardiovascular responses in control and MI rats

**Figure 2:**
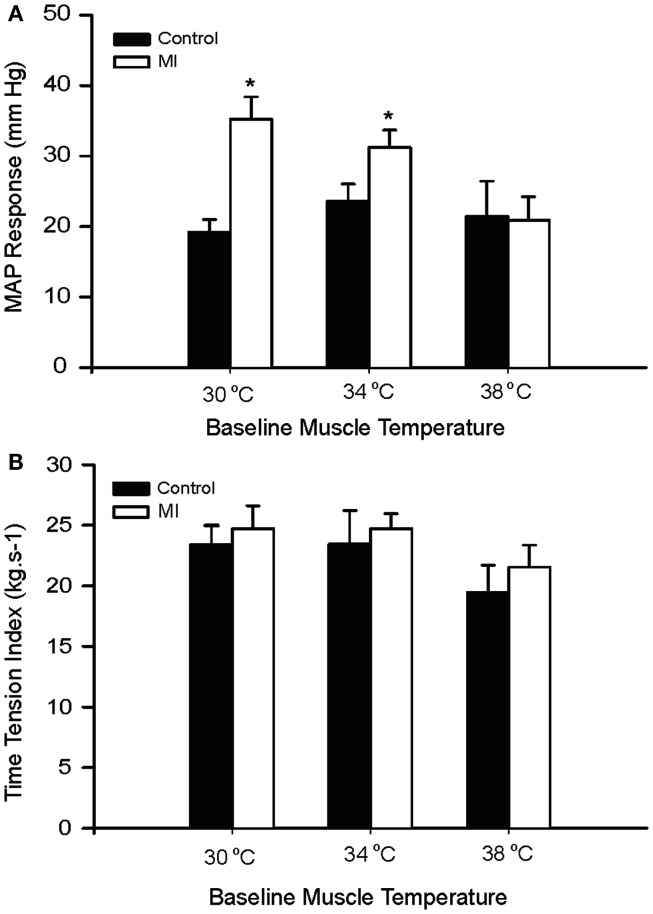
**Peak pressor response and developed muscle tension evoked by sciatic nerve stimulation at three different basal *T*_m_: 30, 34, and 38°C in control and MI rats**. **(A)** MAP responses were significantly augmented during static muscle contraction in MI group at baseline *T*_m_ of 30 and 34°C. **(B)** Developed tensions are indicated by time-tension index (TTI), and the TTI induced by static contraction were similar in two groups. *P < 0.05, compared with healthy control group. The number of rats = 9 in control; and 10 in MI.

Baseline MAP values before contraction were not different in the healthy control animals and the MI rats (Table 3). Electrical stimulation of the sciatic nerve significantly increased MAP in both groups. A greater MAP increase was observed in MI rats, as baseline *T*_m_ was 30 and 34°C but not 38°C (Figure 2A). There was no significant difference in HR response to the stimulation (Table 3).

**Table 3:** **Baseline MAP (mm Hg) and HR (beats/min), and peak responses**.

Baseline *T*_m_	Groups	Baseline MAP	Peak MAP	Baseline HR	Peak HR
30°C	Control	80 ± 3	99 ± 4*	390 ± 13	410 ± 13
	HF	84 ± 5	119 ± 6*	399 ± 16	423 ± 17
34°C	Control	94 ± 6	117 ± 7*	390 ± 23	414 ± 25
	HF	93 ± 6	124 ± 7*	418 ± 20	443 ± 20
38°C	Control	88 ± 7	109 ± 11*	400 ± 16	406 ± 17
	HF	94 ± 6	114 ± 8*	422 ± 20	432 ± 24

Values are means ± SE. The number of animals = 9 in control; and 10 in HF. MAP, mean arterial pressure; HR, heart rate; *T*_m_, muscle temperature. There are no significant differences among basal values. *P < 0.05, versus baseline.

It is noted that there were no significant differences in the muscle tension indicated by time-tension index in the two groups (Figure 2B). This suggests that attenuated *T*_m_ and enhanced pressor responses to contraction in the MI rats were not due to development of muscle tension. In addition, absolute muscle tensions appeared to be smaller in MI rats; however, no significant differences were observed in both groups. When baseline *T*_m_ was 30, 34, and 38°C, peak tensions were 0.55 ± 0.04, 0.57 ± 0.06, and 0.46 ± 0.04 kg in control; and 0.52 ± 0.03, 0.51 ± 0.02, and 0.44 ± 0.03 kg in MI (*P* > 0.05, vs. control at all conditions).

### A relationship between pressor and *T*_m_ response

**Figure 3:**
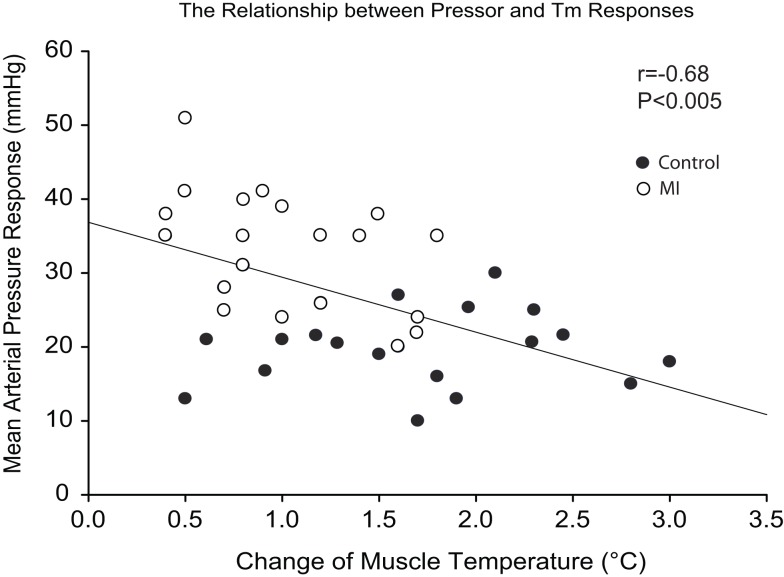
**Regression analyses between MAP response and muscle temperature change during static contraction at baseline *T*_m_ of 30 and 34°C**. Note that a higher MAP response was seen as a lower *T*_m_ was induced by muscle contraction.

Changes in MAP response to static muscle contraction were plotted against contraction-induced increase in *T*_m_. A significant inverse correlation was seen between the pressor response and increased *T*_m_ during contraction (Figure 3). Thus, the greater pressor response is likely linked to the lower *T*_m_ response in rats with MI.

## Discussion

Our data demonstrate that contraction-induced increase in *T*_m_ is attenuated in MI rats compared with control rats. A regression analysis further shows that the lower *T*_m_ response is closely related to the greater pressor response during contraction. Notably, there are no differences in the muscle tension development and muscle mass in the control rats and rats with HF. This suggests that altered *T*_m_ and pressor responses in HF are unlikely due to muscle tension and/or muscle mass. Additionally, body core temperature is unchanged during contraction, indicating that body temperature is unlikely to affect the *T*_m_ and pressor responses. Thus, findings of this study suggest that impaired *T*_m_ response is likely responsible for the exaggerated muscle pressor reflex in HF.

### Effects of sensory nerves’ P2X and TRPV1 on muscle pressor reflex

Evidence supports that ATP sensitive P2X receptors play a role in evoking mechano- and metaboreceptors mediated stimulation of the exercise pressor reflex (Hanna et al., 2002; Li and Sinoway, 2002; Hanna and Kaufman, 2003, 2004). In HF, P2X plays a role in sympathetic abnormalities by demonstrating that the muscle pressor response induced by α, β-me ATP injected into rat hindlimb muscles is exaggerated in HF animals (Gao et al., 2007). Also, P2X receptor-mediated muscle mechanoreceptor contribution to the sympathetic nerve response to exercise is augmented in HF (Li et al., 2004b; Wang et al., 2010). Moreover, the greater response to activation of PX receptor is related to the severity of left ventricular dysfunction (Gao et al., 2007). This may be due to that HF induces upregulation of P2X receptors in the sensory neurons (Gao et al., 2007). However, the intrinsic mechanism responsible for the P2X receptor alternations in rats with HF is unclear.

It has been reported that P2X receptors are temperature-sensitive and P2X activity increases as temperature falls (Garcia-Villalon et al., 1997; Ziganshin et al., 2002; Kluess et al., 2005). The effect of P2X receptor on reflex muscle response has recently been reported to be sensitive to alternations of *T*_m_ (Gao et al., 2006). This prior study further suggests that elevated *T*_m_ attenuates the pressor response to static muscle contraction.

Result of the present study shows that *T*_m_ and pressor responses during contraction tended to be smaller in both healthy and MI rats as baseline *T*_m_ was set at 38°C. Moreover, the greater pressor response was linearly related to the lower *T*_m_ response. This is consistent with the concept that the effect of P2X receptor on reflex muscle response is sensitive to alternations of *T*_m_ and that elevated temperature attenuates the response (Gao et al., 2006). In addition, our data also show that an increase in *T*_m_ during contraction was attenuated in HF rats compared with control rats. Thus, the enhanced P2X activity and exaggerated muscle reflex in HF is likely attributed to a lower *T*_m_ response.

Metabolite sensitive-TRPV1 receptors have been reported to respond to heating (Wang et al., 2003). In this report, a rise in *T*_m_ in contracting muscle was smaller in the MI rats than in the controls. Thus, we postulate that the muscle metaboreflex is blunted by less stimulation of TRPV1 due to lower temperature response in HF. A lower *T*_m_ in HF is likely to contribute to the attenuated TRPV1 activities. It has previously been reported that TRPV1 of sensory neurons is downregulated and TRPV1 response is attenuated in HF rats (Li et al., 2004b; Smith et al., 2005; Wang et al., 2010). Data of the present report provide further evidence that suggest that abnormal autonomic adjustments to exercise may be due to abnormalities in *T*_m_ response in HF. We believe that in HF mechanoreceptor contribution to muscle sympathetic nerve activity is augmented, whereas metaboreceptor engagement is attenuated. Overall, mechanosensitive afferents may contribute to a greater degree and muscle sympathetic response is exaggerated in HF (Sterns et al., 1991; Middlekauff et al., 2000, 2001; Li et al., 2004b; Momen et al., 2004; Smith et al., 2005).

### Muscle temperature and muscle pressor reflex

It is known that *T*_m_ rises with exercise (Shellock et al., 1985; Kenny et al., 2003). During exercise, *T*_m_ observed in HF patients is abnormal as compared with healthy subjects (Shellock et al., 1985). Cold temperature increases cardiac demand in response to exercise and significantly reduces maximal exercise capacity in patients with HF (Juneau et al., 2002). A further study suggests that warming of exercising legs improves exercise capacity in patients with cardiac disease and low exercise tolerance (Yamanouchi et al., 1996).

An elevation of *T*_m_ by heating muscles increases reflex blood pressure and sympathetic nerve responses to exercise and cooling muscle delays those reflex activities (Ray and Gracey, 1997; Ray et al., 1997). It is noted that *T*_m_ is increased >4°C and decreased >7°C. When *T*_m_ is altered largely, this could directly change sympathetic nervous activity. Also, afferent inputs from skin could be activated in these human studies. This may lead to different patterns of sympathetic responses than pure muscle afferent activation. In contrast, in our current report the skin covering the triceps surae muscle and femoral region was surgically separated from the muscle below, thus we have eliminated inputs from cutaneous afferents in the limb.

It should be noted that in the present report the effects of muscle blood flow on changes of *T*_m_ are not determined as muscle contraction is evoked at different temperature conditions. However, previous studies have demonstrated that blood flow directed to exercising muscles is decreased in HF (Lejemtel et al., 1986; Shoemaker et al., 1999), suggesting that blood flow is likely to contribute to attenuated *T*_m_ increase in HF as contraction is induced.

Finally, static contraction was induced by the sciatic nerve stimulation in the current study to optimize *T*_m_ and pressor responses. A study limitation is that the pressor response induced by the stimulation was possibly due, in part, to direct electrical activation of afferent nerves. However, contraction-induced increase in *T*_m_ was unlikely due to the direct stimulation of afferent nerves because core temperature and *T*_m_ on the contralateral leg were not altered. Also, the pressor response was closely related to change of *T*_m_ during contraction. This is important to address the issue that impaired *T*_m_ response can modify the muscle reflex in HF.

In summary, the data of present study demonstrate that contraction-induced increase in *T*_m_ is attenuated in HF animals compared with control animals, and the magnitude of the pressor response to contraction is greater as the temperature increase is lower. The impaired temperature response may alter muscle afferent-mediated pressor response via temperature-sensitive P2X and TRPV1 receptors. This investigation provides evidence for the role played by *T*_m_ in the exaggerated sympathetic responses to exercise in HF.

## Conflict of Interest Statement

The authors declare that the research was conducted in the absence of any commercial or financial relationships that could be construed as a potential conflict of interest.
